# Reduced Glutathione suppresses Oxidative Stress in Nonalcoholic Fatty Liver Disease

**DOI:** 10.5005/jp-journals-10018-1159

**Published:** 2016-07-09

**Authors:** Makoto Irie, Tetsuro Sohda, Akira Anan, Atsushi Fukunaga, Kazuhide Takata, Takashi Tanaka, Keiji Yokoyama, Daisuke Morihara, Yasuaki Takeyama, Satoshi Shakado, Shotaro Sakisaka

**Affiliations:** 1 Department of Gastroenterology and Medicine, Fukuoka University, Faculty of Medicine, Fukuoka, Japan

**Keywords:** 8-Hydroxy-2-deoxyguanosine, Gamma-glutamyltranspeptidase, Glutathione, Nonalcoholic fatty liver disease, Nonalcoholic steatohepatitis.

## Abstract

**Background and aims:**

Insulin resistance and cytokine production are key mechanisms leading to fatty change in the liver and may produce nonalcoholic steatohepatitis (NASH). Oxidative stress may also contribute to clinical progression from simple fatty liver (FL) to NASH. A therapy for insulin resistance and antioxidant has been applied to treat NASH, yet these treatments are not fully established. In the present study, we have evaluated whether an antioxidant agent, glutathione, prevents the development of NASH from FL.

**Materials and methods:**

Five patients with FL and 10 with NASH were enrolled in the study. Three hundred milligrams per day of glutathione was given orally to patients with nonalcoholic fatty liver disease (NAFLD) every day, and an oxidative stress marker and biochemical tests were analyzed before treatment and 1 and 3 months after starting the treatment. We measured serum levels of 8-hydroxy-2-deoxyguanosine (8-OHdG) and gamma-glutamyltranspeptidase (GGT). Immunohistochemistry for glutathione was performed on formalin fixed liver specimens obtained from liver biopsies.

**Results:**

Before treatment, the NASH group had higher serum 8-OHdG and lower serum glutathione levels than the FL group. Immunohistochemistry revealed that a strong expression of glutathione was observed in zone 3 in both NASH and FL before treatment. Serum levels of alanine transaminase and 8-OHdG were significantly decreased after treatment in the NASH group. Gamma-glutamyltranspeptidase was decreased after treatment, although the decrease was statistically not significant.

**Discussion:**

The present pilot study demonstrated that antioxidant therapy with glutathione may reduce the pathological oxidative stress in the liver in NASH, preventing the progression from NAFLD to NASH.

**How to cite this article:**

Irie M, Sohda T, Anan A, Fukunaga A, Takata K, Tanaka T, Yokoyama K, Morihara D, Takeyama Y, Shakado S, Sakisaka S. Reduced Glutathione suppresses Oxidative Stress in Nonalcoholic Fatty Liver Disease. Euroasian J Hepato-Gastroenterol 2016;6(1):13-18.

## INTRODUCTION

Nonalcoholic fatty liver disease (NAFLD) is one of the most common chronic liver conditions worldwide and is characterized by fat accumulation in the absence of significant alcohol consumption and other causes of liver disease, such as medications or other disease processes.^1^ The severity of disease ranges from simple steatosis to steatohepatitis (NASH) and can eventually lead to cirrhosis or hepatocellular carcinoma.^2-4^ Recent studies have suggested that oxidative stress may also contribute to clinical progression from fatty liver (FL) to NASH.^5^ Markers used to assess oxidative stress include malondialdehyde (MDA) and 4-hydroxy-2-nonenal (4HNE), a product of lipid peroxidation, and 8-hydroxy-2-deoxyguanosine (8-OHdG), a product of oxidative DNA damage mainly by hydroxyl radicals.^6^ Intrahepatic 8-OHdG expression has been shown to be increased in NASH compared with FL and normal liver, with 8-OHdG being particularly expressed in areas of active inflammation.^7^

The primary role of hepatocellular gamma-glutamyltranspeptidase (GGT) is the metabolism of extracellular reduced glutathione, allowing precursor amino acids to be assimilated and reused for intracellular glutathione synthesis.^8^ In oxidative stress, the serum level of oxidized glutathione increases and hepatic GGT is induced; this then dismantles the oxidized glutathione and converts it to reduced glutathione. Gamma-glutamyltranspeptidase, therefore, has an important role in antioxidant defense systems at the cellular level and is a valuable marker of oxidative stress in NAFLD.^9,10^ We have reported that levels of GGT in FL patients may compensate for mild oxidative stress by repressing 8-OHdG levels and preventing progression to NASH; oxidative stress leads to increased levels of 8-OHdG and the development of NASH.^11^ It may also contribute to clinical progression from simple FL to NASH.

Glutathione is the most abundant cellular thiol antioxidant and exhibits numerous and versatile functions. Oxidative stress occurs when reactive oxygen species (ROS) are produced at levels exceeding those capable of being sequestered by normal cellular antioxidant defenses.

Weight control achieved by diet and exercise is the most important aspect of treatment in obese patients with NAFLD, including NASH. There have been several reports of therapeutic effects on NASH using metformin and thiazolidine derivatives, or colestimide and α-tocopherol.^12,13^ However, these studies are considered to lack the number of study subjects or the control study. A therapy on patients with insulin-resistance and antioxidant have been introduced for NASH, but these are not yet established. In the present study, we aim to prevent the development of NASH from FL by antioxidant therapy.

## MATERIALS AND METHODS

### Patients and Methods

Five patients with FL and 10 with NASH, diagnosed on the basis of percutaneous liver biopsy between April 2010 and March 2014, were enrolled into the present study. Patients positive for hepatitis B surface antigen or hepatitis C virus and those < 20 years of age were excluded from the study. Three hundred milligrams per day of glutathione was given orally to patients with FL and NASH, daily.

### Measurement of Serum 8-OHdG and GGT Levels

We measured serum 8-OHdG and GGT as previously described.^11^ In brief, serum levels of 8-OHdG were measured using the highly sensitive 8-OHdG Check enzyme-linked immunosorbent assay (ELISA) kit (Nikken Zail, Shizuoka, Japan) according to the manufacturer’s instructions. Serum levels of GGT were measured using the Qualigent® GGT kit (Sekisui Medical, Tokyo, Japan). Serum levels of glutathione (GSH) were measured using the GSH kit (Nikken Zail, Shizuoka, Japan).

### Immunohistochemistry

Immunohistochemistry for GSH was performed on formalin fixed liver specimens obtained from liver biopsies as previously described.^14^ In brief, liver tissue was immunostained with an antibody of an anti-glutathione S-transferase alpha antibody (glutathione, dilution 1:300; Abcam, Tokyo, Japan). Peroxidase activity was detected using a liquid diaminobenzidine (DAB) substrate kit (Histofine® DAB-3S kit, Nichirei Biosciences, Tokyo, Japan). The slides were viewed using a conventional light microscope (Carl Zeiss, Germany) at × 200 magnifications.

### Statistical Analyses

The mean ± SD was calculated for each set of data. Comparisons between the groups were performed using the student’s t-test. A p-value < 0.05 was considered to be statistically significant. All statistical analyses were performed using JMP® software version 11 (SAS Institute Inc., Tokyo, Japan).

### Ethics

The study was conducted according to the principles of the Declaration of Helsinki and the protocol was approved by the Ethics Committee of Fukuoka University Hospital. All patients gave written informed consent prior to their participation.

## RESULTS

**Figs 1A to C:**
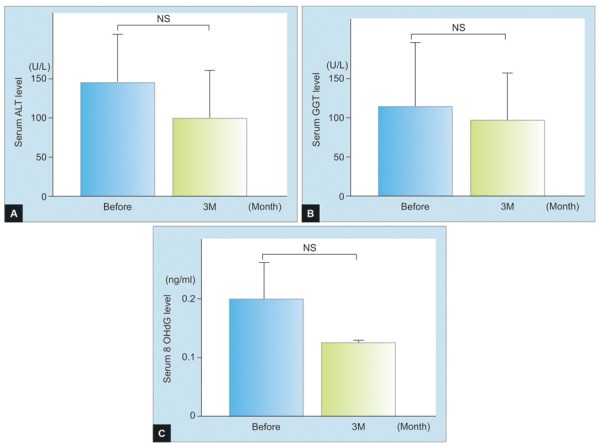
(A) Serum levels of alanine aminotransferase, (B) γ-glutamyltranspeptidase, and (C) 8-hydroxy-2-deoxyguanosine; and in fatty liver; NS: Not statistically significant; student’s t-test

**Figs 2A to C:**
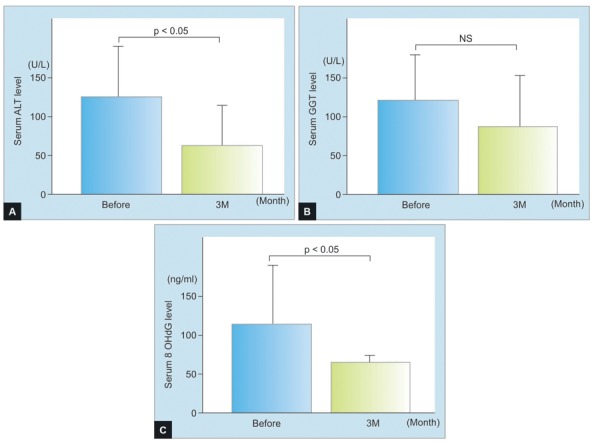
(A) Serum levels of alanine aminotransferase, (B) γ-glutamyltranspeptidase, and (C) 8-hydroxy-2-deoxyguanosine in nonalcoholic steatohepatitis, NS: Not statistically significant; student’s t-test

**Fig. 3:**
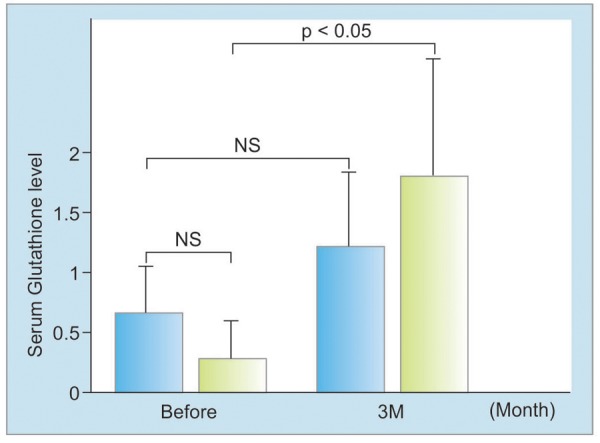
Serum glutathione levels in FL and NASH; NS: Not statistically significant; student’s t-test

Five patients with FL and 10 with NASH were recruited to the study and their demographic, clinical, and biochemical characteristics taken, as given in Table 1. Serum levels of ALT, GGT, and 8-OHdG were decreased after treatment in FL, although these were not statistically significant (Figs 1A to C). Serum levels of ALT and 8-OHdG, but not GGT, were significantly decreased in NASH after treatment (p < 0.05) (Figs 2A to C). Before treatment, serum glutathione levels were higher in FL than in NASH, which increased significantly by 3 months’ treatment (p < 0.05) (Fig. 3).

**Table 1:** **Table 1:** Clinical parameters

*Characteristic*		*Fatty liver (n = 5)*		*Nonalcoholic steatohepatitis (n = 10)*		*Statistical significance*	
Age, years		38 ± 17		62±9		< 0.05*	
Gender (males/females)		4/1		3/7			
Body mass index (kg/m^2^)		25.9 ± 3.5		26.5 ± 3.0		NS	
Albumin (g/dL)		4.8 ± 0.4		4.5 ± 0.6		NS	
Total bilirubin (mg/dL)		1.1 ± 0.9		0.8 ± 0.2		NS	
Aspartate transaminase (U/l)		59.4 ± 25.4		63.6 ± 39.5		NS	
Alanine transaminase (U/l)		146 ± 87		125 ± 74		NS	
Alkaline phosphatase (U/l)		298 ± 51		306 ± 172		NS	
γ-Glutamyltranspeptidase (U/l)		97 ± 65		120 ± 75		NS	
Total cholesterol (mg/dL)		228 ± 50		225 ± 47		NS	
Triglycerides (mg/dL)		132 ± 24		184 ± 112		NS	
Glucose (mg/dL)		103 ± 5.6		142 ± 43		NS	
HbA1c (%)		5.6 ± 1.6		6.8 ± 1.2		< 0.05*	
Insulin (μU/mL)		17 ± 4		18 ± 15		NS	
HOMA-R		4.2 ± 0.9		6.1 ± 6.0		NS	
Ferritin (ng/mL)		225 ± 77		243 ± 124		NS	
Hyaluronic acid (ng/mL)		12 ± 2		81 ± 60		< 0.05*	

All data expressed as the mean ± SD. p value calculated using Student t-test. NS: Not significant; HbA1c: Glycosylated hemoglobin; HOMA-R: Homeostasis model assessment insulin resistance.

*Statistical difference between the fatty liver and nonalcoholic steatohepatitis.

**Figs 4A and B:**
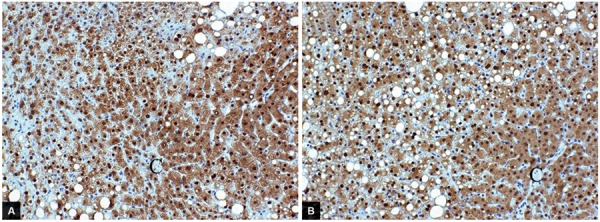
Immunohistochemical staining of glutathione in liver tissues. Immunoreactivity glutathione was strongly expressed in the cytoplasm of hepatocytes in an FL tissue; (A) as well as in a NASH tissue; (B) in the liver lobule zones 3 (× 200). There were no differences in the localization, but immunoreactivity of glutathione expression is stronger in an FL tissue than in an NASH tissue; and (C) Central vein

Immunohistochemistry revealed that a strong expression of glutathione was observed in zone 3 in FL, and immunoreactivity of glutathione was expressed stronger in FL than in NASH (Figs 4A and B).

## DISCUSSION

Westernization of the Japanese lifestyle has led to increase interests among the Japanese population in lifestyle-related diseases. But increased incidences of obesity and of metabolic syndrome and its associated insulin resistance, hyperlipidemia, and hypertension are also seen in Japan.

Elucidation of the mechanisms underlying the progression of FL to NASH within the spectrum of NAFLD, the hepatic manifestation of metabolic syndrome, is important; insulin resistance,^15^ oxidative stress,^16^ endotoxins,^17^ and inflammatory cytokines^18^ may all be involved, with the inflammation and fibrosis caused by these various liver-damaging factors leading to the development of NASH. Oxidative stress occurs when there is an imbalance between defense factors, such as superoxide dismutase and reduced glutathione, and ROS such as superoxide.^19^ Depletion of antioxidants such as glutathione, vitamin E, β-carotene, or vitamin C and/or increased production of reactive oxygen species in the liver may occur in NASH. Serum oxidative stress is known to contribute to liver cirrhosis, which is a clear risk factor for hepatocellular carcinoma.^20^ Oxidative stress markers, such as serum levels of 8-OHdG or 4HNE, have been reported to be raised and antioxidants such as superoxide dismutase decreased in patients with NASH; however, no significant difference in serum 8-OHdG levels between NASH and FL was reported.^21^ The enzyme GGT pays an important role in reducing oxidized glutathione during oxidative stress.^14^ In oxidative stress, oxidized glutathione increases and hepatic GGT is induced; this then dismantles oxidized glutathione and converts it to reduced glutathione. Thus, hepatic GGT is thought to defend against liver and DNA damage caused by oxidative stress. Glutamine reduces the degree of oxidative stress and improves hepatic steatosis in the livers of rats with NAFLD. Glutamine reduces the degree of oxidative stress and improves hepatic steatosis in the livers of rats with NAFLD.^22^ In the present study, serum glutathione levels increased both in FL and in NASH 3 months after glutathione treatment. In the present study, the expression of GSH in liver tissue is abundant in FL and moderately localized in NASH. However, serum GSH levels in NASH groups decreased (Fig. 3). It has been reported that supplementation of N-acetylcysteine (NAC), an antioxidant that increases tissue GSH levels by supplying cysteine for GSH synthesis, is able to rescue hepatic fibrosis mice into adulthood.^23^ Antioxidant therapy decreases serum levels of ALT and 8-OHdG in NASH.

**Fig. 5:**
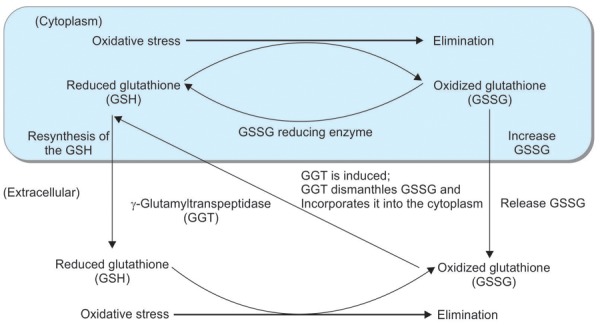
The role of GGT for the oxidative stress in NAFLD. Glutathione is a strong scavenger present in the liver. When glutathione is depleted, GGT is thought to be induced. By reducing oxidative stress, the glutathione may prevent the progression of liver damage in NASH

Reduced oxidative stress, and the presence of glutathione in NASH, may prevent further liver damage. Therefore, glutathione could be an important and promising therapeutic drug for the prevention of NASH progression. The role of GGT for the oxidative stress may indicate the progression of NASH from NAFLD. Glutathione is a strong scavenger present in the liver, and when glutathione is depleted, it is thought that GGT levels increase. By reducing oxidative stress, the glutathione treatment for NASH may control the progression of liver damage (Fig. 5). In conclusion, the present pilot study demonstrated that antioxidant therapy may prevent the progression of NAFLD to NASH through reduction of oxidative stress.
